# Supplementary material for: Betanin purification from red beetroots and evaluation of its anti-oxidant and anti-inflammatory activity on LPS-activated microglial cells
Source: PLoS One. 2020 May 13;15(5):e0233088. doi: 10.1371/journal.pone.0233088 (PMC7219731; doi:10.1371/journal.pone.0233088)
Supplement: Attachment — Submitted filename: Response to Reviewers.docx [file pone.0233088.s001.docx]

Editor Comments (if provided):

1. We understand that you purchased red beetroots from your local
market for this study. In your Methods section, please provide additional details regarding the source of this material. Please provide the geographic coordinates and names of the purchase location. if available, as well as any further details about the purchased items (e.g., lot number, source origin, description of appearance) to ensure reproducibility of the analyses.

Related explanation was added to the Materials section

The authors should check the effect of Betanin on NF-kB activation as suggested by Reviewer#2.

Response:

We tried to perform more experiments as you suggested but due to the Coronavirus outbreak, the laboratories are still closed, and also having access to materials for such experiments will take 2-3 months in this new situation. However, we added more explanation from the results already published in the literature that may be a proper response for this question as below (same explanation was also added to the manuscript).

In LPS-activated macrophages, p38 immediately phosphorylated at a tyrosine residue and performs a crucial role in inflammation . Activating of p38 MAPKs is involved in the upregulation of inflammatory mediators, including TNF-α, iNOS, and cyclooxygenase-2 (COX-2) (1). On the other hand, activation of the p38 and NF-κB pathways could be accomplished by the TNFR1 and TNF-α (2). The obtained results of attenuating in the expression of Tnf-α and Nos2 at mRNA level, TNF-α protein, and the product of iNOS (*NO^•^*), could be attributed to either the direct effect of p38 on these factors or the negative effect of betanin on the performance of p38.

The discussion section should be improved by discussing relevant literature such as PMID: 31315033; PMID: 25083991; PMID: 31085174; PMID: 18566231 etc.

This section was improved by adding more discussion accordingly.

**Comments to the Author**

**Reviewer #1:**
I have one major issue which is with the resolution and presentation of Figure-6 which is docking figure. Author should make it more visible and easily understandable while adding appropriate leveling and also mention values for negative binding energy

Response:

Thank you for your comment. Modification on all figures including Figure 6 was made to improve their qualities.

**Reviewer #2:**
1. Figures are not well-prepared. The resolution of all the figures is very poor.

Response:

The quality of all the figures were improved according your suggestion

2. For FACS staining of microglial cells, combination of CD11b and CD45 labelling will be more proper otherwise it’s very hard to distinguish microglia from macrophages. Alternatively, specific microglial marker MEM119 can be used.

Response:

We use CD11b as the most useful immunohistochemical marker for microglial cells in healthy brains (3–5). Similarly, several studies have also used cd11b alone to identify microglial cells especially in in-vitro studies like the primary culture of microglial cells (6–9). However, more specific microglial marker TMEM119 could be used to distinguish microglial cells from other immune cells (10).

3. For its anti-inflammatory activity on LPS-activated microglial cells, the author should also validate by checking phospho-NF-κB p65. As another master regulator of inflammation, does p38 signalling also involve in betamin mediated anti-inflammatory activity?

Response:

We tried to perform more experiments as you suggested but due to the Coronavirus outbreak, the laboratories are still closed, and also having access to materials for such experiments will take 2-3 months in this new situation. However, we added more explanation from the results already published in the literature that may be a proper response for this question as below (same explanation was also added to the text).

In LPS-activated macrophages, p38 immediately phosphorylated at a tyrosine residue and performs a crucial role in inflammation (11). Activating of p38 MAPKs is involved in the upregulation of inflammatory mediators, including TNF-α, iNOS, and cyclooxygenase-2 (COX-2) (1). On the other hand, activation of the p38 and NF-κB pathways could be accomplished by the TNFR1 and TNF-α (2). The obtained results of attenuating in the expression of Tnf-α and Nos2 at mRNA level, TNF-α protein, and the product of iNOS (*NO^•^*), could be attributed to either the direct effect of p38 on these factors or the negative effect of betanin on the performance of p38.

1.	Yi YS, Son YJ, Ryou C, Sung GH, Kim JH, Cho JY. Functional roles of Syk in macrophage-mediated inflammatory responses. Mediators Inflamm. 2014;2014.

2.	Blaser H, Dostert C, Mak TW, Brenner D. TNF and ROS Crosstalk in Inflammation. Trends Cell Biol. 2016 Apr;26(4):249–61.

3.	Hickstein DD, Ozols J, Williams SA, Baenziger JU, Locksley RM, Roth GJ. Isolation and characterization of the receptor on human neutrophils that mediates cellular adherence. J Biol Chem. 1987;262(12):5576–80.

4.	Sasaki A. Microglia and brain macrophages: An update. Neuropathology. 2017;37(5):452–64.

5.	Jeong H-K, Ji K, Min K, Joe E-H. Brain Inflammation and Microglia: Facts and Misconceptions. Exp Neurobiol. 2013;22(2):59–67.

6.	Lacagnina MJ, Fabisiak TJ, Grace PM. Mapping microglial reactivity in the brain after sciatic nerve injury. Brain Behav Immun [Internet]. 2017;66(2017):e16. Available from: https://doi.org/10.1016/j.bbi.2017.07.068

7.	Morrison HW, Filosa JA. Sex differences in astrocyte and microglia responses immediately following middle cerebral artery occlusion in adult mice. Neuroscience [Internet]. 2016;339:85–99. Available from: http://dx.doi.org/10.1016/j.neuroscience.2016.09.047

8.	Hutter-Schmid B, Humpel C. Primary mouse brain pericytes isolated from transgenic Alzheimer mice spontaneously differentiate into a CD11b+ microglial-like cell type in vitro. Exp Gerontol [Internet]. 2018;112(July):30–7. Available from: https://doi.org/10.1016/j.exger.2018.08.003

9.	Jia X, Cong B, Wang S, Dong L, Ma C, Li Y. Secondary damage caused by CD11b+ microglia following diffuse axonal injury in rats. J Trauma Acute Care Surg. 2012;73(5):1168–74.

10.	Bennett ML, Bennett FC, Liddelow SA, Ajami B, Zamanian JL, Fernhoff NB, et al. New tools for studying microglia in the mouse and human CNS. Proc Natl Acad Sci U S A. 2016;113(12):E1738–46.

11.	Lee JC, Laydon JT, McDonnell PC, Gallagher TF, Kumar S, Green D, et al. A protein kinase involved in the regulation of inflammatory cytokine biosynthesis. Vol. 372, Nature. 1994. p. 739–46.
